# An X-STRs analysis of the Iraqi Sorani Kurds

**DOI:** 10.1371/journal.pone.0294973

**Published:** 2023-11-27

**Authors:** Balnd M. Albarzinji, Shams Hadi, Bahez Ismael, Ahmed Barqee, Abdullah Hadi, Hayder Lazim

**Affiliations:** 1 Kurdistan Institution for Strategic Studies and Scientific Research (KISSR), Sulaymaniyah, Iraq; 2 University of Central Lancashire Medical School, Preston, United Kingdom; 3 Faculty of Health, Social Care and Medicine (FHSCM), School of Medicine, Edge Hill University, Ormskirk, United Kingdom; Universiti Teknologi Malaysia - Main Campus Skudai: Universiti Teknologi Malaysia, MALAYSIA

## Abstract

A database for the Iraqi Sorani Kurds, specifically focused on the 12 X-short tandem repeat (STR) loci, has been developed to fascilitate forensic and population genetics investigations. The present study involved genotyping 117 unrelated individuals from the Sorani Kurds ethnic group using the Investigator Argus X-12 QS kit. The analysis revealed that the DXS10135 locus exhibited the highest degree of polymorphism, as indicated by a polymorphism information content (PIC) value of 0.94565 and a gene diversity (GD) value of 0.95623. Conversely, the DXS8378 locus displayed the lowest level of polymorphism, with a PIC value of 0.61026 and a GD value of 0.68170. Notably, two individuals were found to possess a rare allele (allele = 6) at the DXS8378 locus, which was not included in the allelic ladder of the kit. Furthermore, a significant linkage disequilibrium (LD) (p < 0.05/117) was observed between the DXS10103 and DXS10101 loci on linkage group 3 (LG3). The ancestral composition of the five primary geographic regions, namely Africa, Middle East, East Asia, Europe, and South America, was determined through the utilization of the $F_{ST}/F_{ST}^{max}$ ratio. The findings of this analysis revealed that the Middle Eastern populations exhibited the lowest $F_{ST}/F_{ST}^{max}$ ratio, measuring at 0.23243, indicating a relatively lower ancestral diversity. Conversely, the European populations showcased the highest $F_{ST}/F_{ST}^{max}$ ratio, measuring at 0.27122, indicative of a greater ancestral diversity within this region. Additionally, the allelic richness indicators, namely distinctive and private alleles, indicated that Africa and the Middle East displayed the highest levels, while Far East Asia exhibited the lowest. This analysis supports the hypothesis of repeated founder effects during outward migrations, as evidenced by both the ancestry variability and the allelic richness. Consequently, the findings of this study have important implications for forensic genetics and population genetics research, particularly in relation to the consideration of genetic predispositions within specific ethnic groups.

## Introduction

In many mammal species, including humans, the X chromosome is one of the two chromosomes that determines sex. It is only found in one copy in males, sharing this characteristic with both the Y chromosome and the mitochondrial DNA (mtDNA) [1].

The estimated length of the X chromosome is 155 million base pairs (Mb), or about 5% of the estimated size of the human genome (3,200 Mb). The X chromosome of the male gender is predominantly transmitted to females as a single unit. In contrast, the two copies in females recombine, like autosomes, thus reorganising genetic variation in each generation and increasing haplotype diversity. The newly rearranged chromosome is then passed on to both male and female offspring [1–4].

In forensic genetics, Short Tandem Repeat (STR) markers on the X and Y chromosomes have been developed as valuable tools that can complement the analysis of autosomal STR and mtDNA [5]. The major advantage of X chromosome short tandem repeats (X-STRs) is the analysis of female traces in male backgrounds and complicated kinship deficiency cases [6]. Moreover, X chromosome markers are also used in population analyses to understand the genetic diversity of a population and consequently can be helpful in demographic studies such as migration [7]. One of the commercial kits that have been used to analyse X-STRs in many populations worldwide is the Investigator Argus X-12 QS kit, which comprises 12 loci organized into four linkage groups (LGs) [8].

The X chromosome is crucial in understanding the differences in population-genetic studies between male and female individuals. Several factors influence the genetic distances found between males and females, including breeding and death rates, polygamy, and patrilocality. Notably, an important aspect contributing to the gender differences is the uneven distribution of the X chromosome, with females harbouring it for twice as long as males. The enormous genetic information encoded inside the X chromosome has the potential to provide crucial insights that cannot be obtained by analysing either the Y chromosome or mtDNA. Despite having substantially more genetic information than the Y chromosome or mtDNA, the X chromosome gets little attention in population genetic studies [1, 3].

Iraq is a nation located in the Arabian Peninsula, with a population of approximately 40 million people. It shares borders with several countries, including the Arabian Gulf, Kuwait, Saudi Arabia, Jordan, Syria, Turkey, and Iran. The population of Iraq consists of five distinct ethnic groups, with the Arabs and Kurds being the predominant groups. The Kurds, who inhabit a mountainous region spanning Iraq, Iran, Syria, Turkey, and Armenia, have different dialects such as Kurmanji, Sorani, Kirmashani, Zazaki, and Gorani. However, there is limited published data available on the comprehensive diversity of the population in Iraq and the genetic diversity of the Kurdish population [9–11]. This study focuses on the Sorani group, which is part of the Central Kurdish group in the northeastern Iraqi province of Sulaymaniyah. The Sulaymaniyah province, with a population of 779,000, is geographically situated amidst Iran to the east and Iraqi Arabs to the south [9].

For the first time, a representative database of X-STRs loci for the central Sorani Kurdish population of northern Iraq was presented to the scientific community. This study also looked at potential demographic differences based on genetic distances and allele frequencies between single populations and continental regions. Further, this study included a global investigation of the X chromosome’s ancestral variability, allelic richness, and various populations’ genetic structures.

## Materials and methods

### Sampling and DNA extraction

Buccal swab samples were collected from 117 Sorani Kurd males aged 18 years old and above. All work in this manuscript was reviewed and approved following institutional ethical guidelines. The Ethics Committee of the Kurdistan Institution for Strategic Studies and Scientific Research/Department of Biology granted ethical approval on February 15, 2020, with a unique reference number (KI-ET 20/February 2020). Informed consent forms were completed by all participants and authors had no access to information that could identify individual participants during or after data collection. The DNA was extracted using a PrimePrep Genomic DNA Extraction Kit from Blood (GeNet Bio-Korea) according to the manufacturer’s protocol [12]. The purity and concentration of the DNA were determined by using an Eppendorf Biophotometer Plus (Eppendorf-Germany) [13]. The data were then collected in September 2020 and made accessible for research purposes in January 2021.

### X-STRs typing

A set of X-STRs genetic markers were amplified using Investigator Argus X-12 QS kit (Qiagen) [14]. Simultaneous amplification was conducted following the manufacturer’s instructions. The markers are distributed along the X chromosome and located in four LGs: LG 1—DXS10148, DXS10135, and DXS8378; LG 2 –DXS7132, DXS10079, and DXS10074; LG 3 –DXS10103, HPRTB, and DXS10101; LG 4—DXS10146, DXS10134, and DXS7423. The amplified fragments were separated on an ABI 3500 Genetic Analyzer, and allele calling was performed with GeneMapper^®^ V.5 software (Thermo Fisher Scientific).

### Statistical analysis

#### Forensic and population genetic parameters

The haplotype frequencies were calculated using GenAIEx 6.5 [15, 16]. The forensics statistics including gene diversity (GD), polymorphism information content (PIC) and match probability (PM) were calculated using the STRAF online tool [17]. Power of discrimination (PD) assessment was conducted for both male and female individuals, alongside the determination of the mean exclusion chance (MEC). The calculation of MEC involved the utilization of various formulas, tailored to specific scenarios. For cases involving paternal deficiency, Krüger’s formula was employed, allowing for the examination of the paternal grandmother as a substitute for the putative father [18]. Kishida’s formula, on the other hand, was applied to trios with a daughter [19]. In instances involving duos comprising a daughter and a putative father, or a son and mother, as well as trios with daughters, Desmarais’ formulas were employed [20]. The results from the aforementioned formulas were yielded using StatsX v1.1 [21]. A pairwise exact test of linkage disequilibrium (LD) was performed for all pairs of loci using STRAF online tool [22]. The population genetic structure in our data was evaluated by the analysis of molecular variance (AMOVA). Molecular data were obtained for the Iraqi Sorani Kurds population using X-STRs based on the Investigator Argus X-12 QS kit (Qiagen) and compared with the worldwide available population data. Arlequin 3.5.2.2 software [23, 24] was used to calculate the average pairwise differences between (PiXY) and within populations (PiX), in addition to the corrected average pairwise difference between populations (PiXY − (PiX + PiY)/2). More specifically, genetic distances between groups of males were quantified by F_ST_ calculations based on X-STRs data and multi-dimensional scaling (MDS) plots. MDS analysis is also used to investigate genetic similarities between populations [25] and to visualize the variances of the genetic differences in X-STRs and between populations. The genetic matrices and the MDS plot were generated using R statistical software version 4.0. The phylogenetic tree was constructed from allele frequency data using POPTREE2 online tool [26] and FigTree software [27].

#### Structure statistical analysis

Population structure was investigated using the program STRUCTURE version 2.3.4 [28, 29] with an admixture model. STRUCTURE is a model-based inference of population structure program in which individuals are grouped into a series of statistical clusters based on their multilocus genotypes. Each person had a membership coefficient linked to each cluster, which represented the proportion of ancestry that is attributable to the respective cluster.

This study looked into the structure of five geographical regions: 11 Middle Eastern countries and populations [30–35], three African regions [36–38], seven South Asian countries and populations [39–44], 17 European regions and populations [32, 34, 45–52] and eight South American regions and populations [53, 54]. STRUCTURE output was processed with STRUCTURE HARVESTER to analyse and display likelihood values across numerous values of K, as well as to determine the number of genetic groupings that best suit the data [55, 56]. Then, using CLUMPP program [57, 58], The multiple replicate analyses of each data set were aligned, and the resulting files were used to create a graph of population Q-matrices for the X-STRs data for 12 markers across 46 subpopulations using the Distruct program [59, 60].

This study also looked at the levels of variation in membership coefficients among individuals who belonged to two or more designated clusters. The FSTruct program was used to investigate membership variability differences between admixed and non-admixed populations [61, 62].

To better understand genetic diversity and population relationships, allele distributions were studied across five metapopulations: Middle East, Africa, Far East Asia, Europe and South America. We evaluated the number of distinct alleles in the population and the number of unique (private) alleles to the population, that are not present in other populations, as two fundamental characteristics for a population at a given locus. Both private and distinct alleles are especially useful when looking for highly variable multiallelic markers like microsatellites in populations. The number of distinct alleles and private alleles was counted using the Allelic Diversity Analyzer (ADZE) Version 1.0 software [63, 64].

## Results

### The Sorani Kurds X-STRs database

Haplotype frequencies for each LG were calculated. There were 105, 81, 79, and 107 haplotypes detected in LG1, LG2, LG3, and LG4, respectively. LG4 was the most informative, with a frequency of 0.00855 for the most common haplotype, whereas LG3 was the least informative in the Iraqi Sorani Kurd males and presented in S1 Table in S1 File. A total number of 159 different alleles for all loci were observed, varying from 29 for DXS10135 to 5 for DXS8378 and DXS7423. Allele frequencies of the 12 X-STRs loci shown in S2 Table (S1 File) and S1 Fig (S2 File). Variant alleles were observed in different samples. An off-ladder allele was observed at locus DXS8378 (two samples), duplicated alleles were found at locus DXS7423 in one individual and a null allele was also found in one individual at locus DXS10079 (S2 Fig in S2 File).

The PIC and the GD showed that the most polymorphic marker in the Iraqi Sorani Kurds was DXS10135 (PIC = 0.94565, GD = 0.95623). This marker had a total of 29 alleles, of which allele 24 was the most frequent (0.11111). While the least polymorphic marker was DXS8378 (PIC = 0.61026, GD = 0.68170), which had a total of 5 alleles, of which allele 11 was the most frequent (0.37607). The PD of males and females, along with different MEC computations, followed the same pattern as the GD and PIC, with the highest values observed in DXS10135 and the lowest values observed in DXS8378. The alleles frequencies and forensic parameters of Iraqi central Sorani Kurd males are shown in S2 and S3 Tables in S1 File. The MEC estimates for the 12 loci in the Iraqi Sorani Kurds ethnic group were all shown in S3 Table in S1 File, including MEC_Kruger, MEC_Kishida, MEC_Desmarais, and MEC_Desmarais_duo.

### Linkage disequilibrium analysis

The linkage disequilibrium results for the 66 pairwise comparisons of the Iraqi Sorani Kurdish population are provided in S4 Table in S1 File. After applying Bonferroni’s correction, the data revealed just one significant LD (p < 0.05/117), which was between the DXS10103 and DXS10101 locus pairs from LG 3 (S3 Fig in S2 File). There was no statistically significant LD between pairings of loci from different LGs.

### Single locus analysis in different populations

A low level of genetic diversity was observed in our study at all 12 X-STRs of the Investigator Argus X-12 QS kit (Qiagen) panel (S4 Fig in S2 File). The locus DXS10135 had the highest gene diversity of 0.94185, while the locus DXS8378 had the lowest gene diversity of 0.68004. Some 406 different alleles were observed in the 6,634 X chromosomes analyzed, with a median number of 32 alleles per marker and a range of 12 (DXS8378) to 61 (DXS10135) as shown in S5 Table (S1 File).

### Populations structure

**Fig 1:**
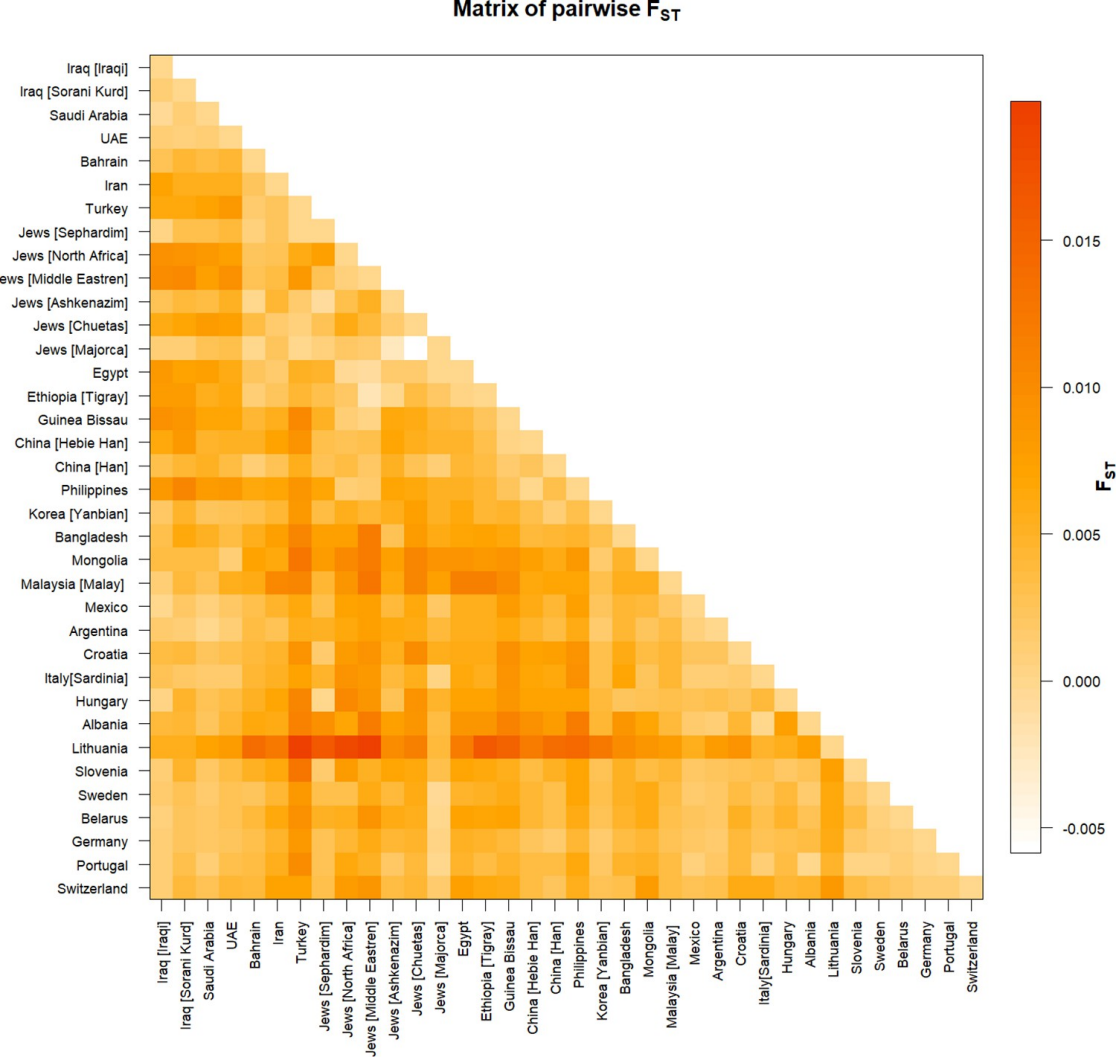
The matrix of pairwise genetic distance F_ST_ of X-STRs 36 populations based on 12 X-STRs markers. This matrix was generated using Arlequin 3.5.2.2 software.

A pairwise matrix plot of F_ST_ distances was generated to compare the 36 populations using 12 loci (Fig 1 and S6 Table in S1 File). The UAE (F_ST_ = 0.00085) had the closest population to Sorani Kurds, while the Philippines (F_ST_ = 0.01053) had the farthest. In the 36 populations studied, the closest association was identified between Jews [Chuetas] and Jews [Majorca] (F_ST_ = -0.0059), and the farthest relationship was identified between Turkey and Lithuania (F_ST_ = 0.0197).

The average pairwise differences were examined to estimate the corrected genetic differences among 36 worldwide populations. These differences were specifically analysed in three contexts: between the 36 populations as a whole, within each individual population, and between different populations using Nei’s distance.

The investigation revealed notable results regarding the average pairwise values obtained by using Nei’s distance. The population of Turkey and the population of Lithuania had the highest average pairwise value, 0.18653. The lowest average pairwise value, -0.05476, was discovered between the Jewish subgroups of Chuetas and Majorca. Furthermore, the Sorani Kurd population had unique average pairwise values. The highest average pairwise value, 0.09900, was found between the Sorani Kurds and the Philippines. The Sorani Kurds and the United Arab Emirates, on the other side, had the lowest average pairwise value (0.00789).

**Fig 2:**
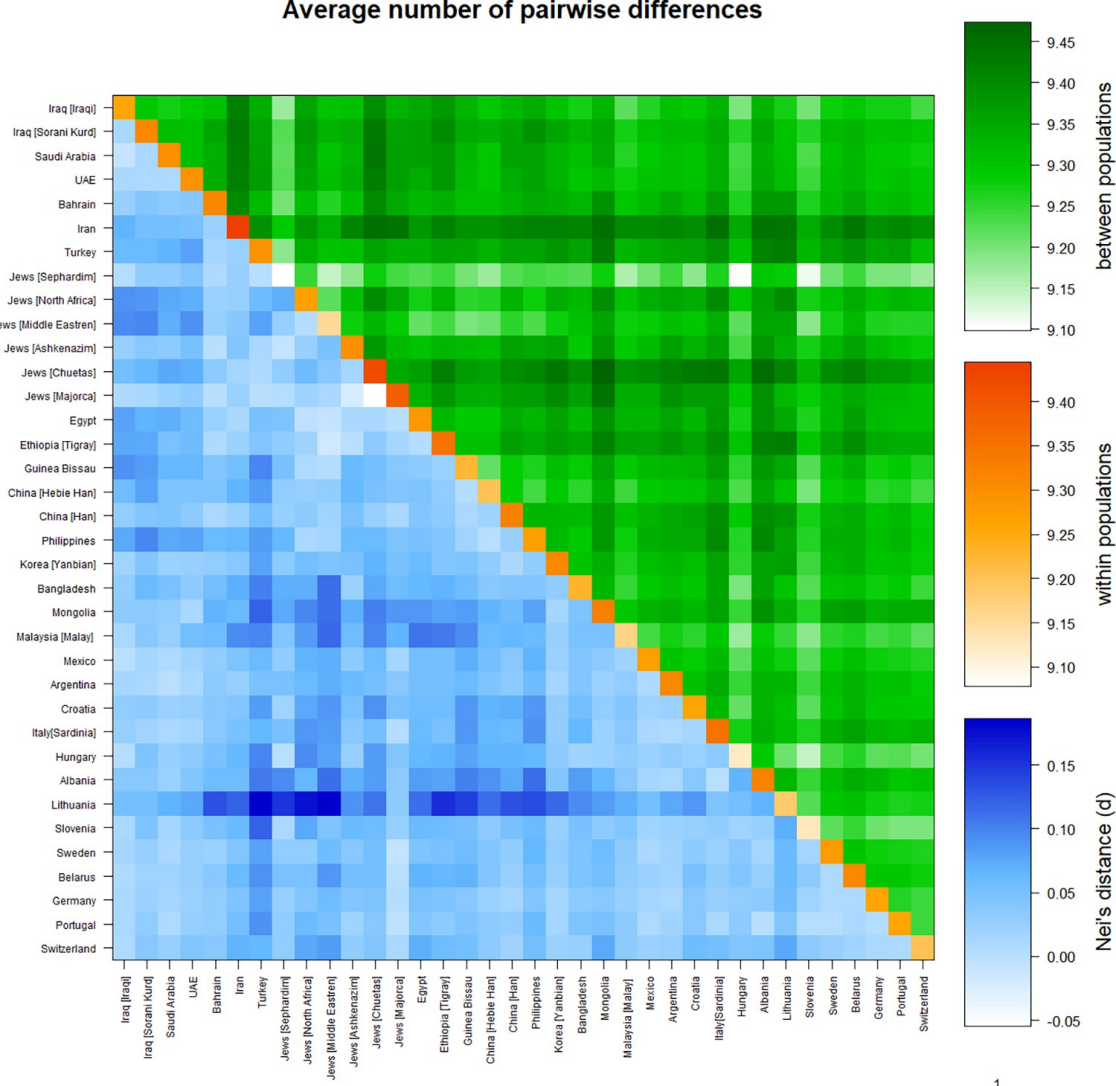
Matrix plot showing population average pairwise differences based on 12 loci and between 36 populations. The area above the diagonal (green) shows the average number of pairwise differences between populations (PiXY); the diagonal (orange) shows the average number of pairwise differences within the population (PiX); and below the diagonal (blue) shows the corrected average pairwise difference (PiXY-(PiX+PiY)/2). The scale of differences is shown on the right side of the matrix. This matrix was generated using Arlequin 3.5.2.2 software.

The Iranian population had the highest average pairwise difference value within populations (9.44449), while Jews [Sephardim] had the lowest (9.07782). Mongolia and Jews [Chuetas] had the highest corrected average pairwise value (9.47319), while Hungary and Jews [Sephardim] had the lowest (9.09849). The Sorani Kurds had the highest corrected average pairwise value (9.43045) with Iran and the lowest (9.22487) with Jews [Sephardim]. The results are shown in S7 Table (S1 File) and Fig 2.

**Fig 3:**
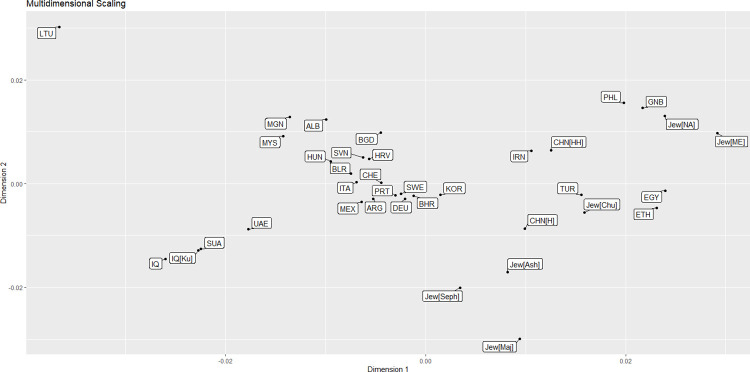
Multidimensional scaling plot of the 36 populations. Population codes are as follows: ALB: Albania; ARG: Argentina; BLR: Belarus; BGD: Bangladesh; BHR: Bahrain; CHE: Switzerland; CHN [H]: China [Han]; CHN [HH]: China [Hebie Han]; DEU: Germany; EGY: Egypt; ETH: Ethiopia; GNB: Guinea Bissau; HRV: Croatia; HUN: Hungary; IRN: Iran; IQ: Iraq; IQ [Ku]: Iraq [Sorani Kurd]; ITA: Italy; Jew [Ash]: Jews [Ashkenazim]; Jew [Chu]: Jews [Chuetas]; Jew [Maj]: Jews [Majorca]; Jew [ME]: Jews [Middle East]; Jew [NA]: Jews [North Africa]; Jew [Seph]: Jews [Sephardim]; KOR: Korea; LTU: Lithuania; MEX: Mexico; MGN: Mongolia; MYS: Malaysia; PHL: Philippines; PRT: Portugal; SUA: Saudi Arabia; SVN: Slovenia; SWE: Sweden; TUR: Turkey; UAE: United Arab Emirates. The MDS fig was generated using R statistical software version 4.0.3.

The MDS was calculated based on F_ST_ genetic distances of 12 X-STRs loci. The resulting MDS plot was generated using R software, depicting the relationships among samples from 36 global populations. The result is shown in Fig 3. The MDS revealed four main clusters, with the central cluster having the most populations (17), followed by clusters of 6 in the upper right quadrant, 8 in the lower right quadrant, and 4 in the lower left quadrant. Along with the populations of Saudi Arabia, the United Arab Emirates, and Iraq, Sorani Kurds are clustered in the lower left quadrant. The sole population that did not exhibit clustering was the population of Lithuania, which was isolated within the upper left quadrant.

**Fig 4:**
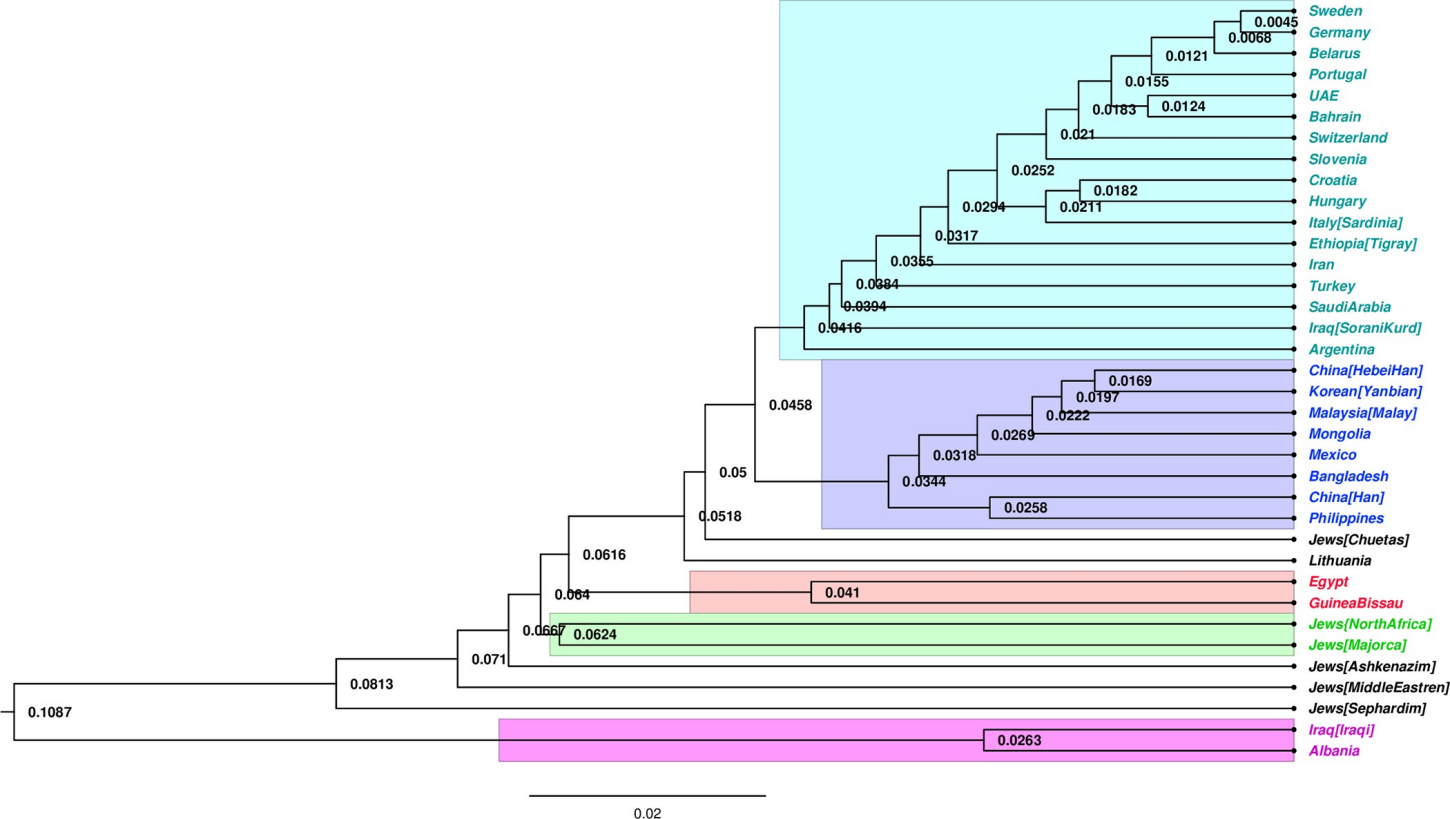
Phylogenic tree of genetic relationships among the 36 global populations. This phylogenic tree was generated using POPTREE2 software. Five clusters (K = 5) were created. Iraqi Sorani Kurds fell into the biggest cluster. The cyan-coloured cluster is the largest (17 populations), followed by the purple-coloured cluster (8 populations), and the remaining clusters each have two populations.

To measure the diversity between populations a phylogenetic tree was constructed based on allele frequency data using POPTREE2 software [14]. FigTree software was subsequently used to visualise the phylogenetic tree. Five subpopulations (K = 5) were the optimal clustering for the 36 global populations (Fig 4). The Iraqi Sorani Kurds were assigned to the largest cluster (cluster 1). The inferred subpopulations were as follows: cluster 1: Sweden, Germany, Belarus, Portugal, UAE, Bahrain, Switzerland, Slovenia, Croatia, Hungary, Italy [Sardinia], Ethiopia [Tigray], Iran, Turkey, Saudi Arabia, Iraq [Sorani Kurd], Argentina, cluster 2: China [Hebei Han], Korean [Yanbian], Malaysia [Malay], Mongolia, Mexico, Bangladesh, China [Han], Philippines, cluster 3: Egypt, Guinea Bissau, cluster 4: Jews [North Africa] and Jews [Majorca], and cluster 5: Iraq [Iraqi], Albania. In addition to Lithuania, four Jewish populations, Chuetas, Ashkenazim, Middle Eastern, and Sephardim, did not demonstrate any clustering.

**Fig 5:**
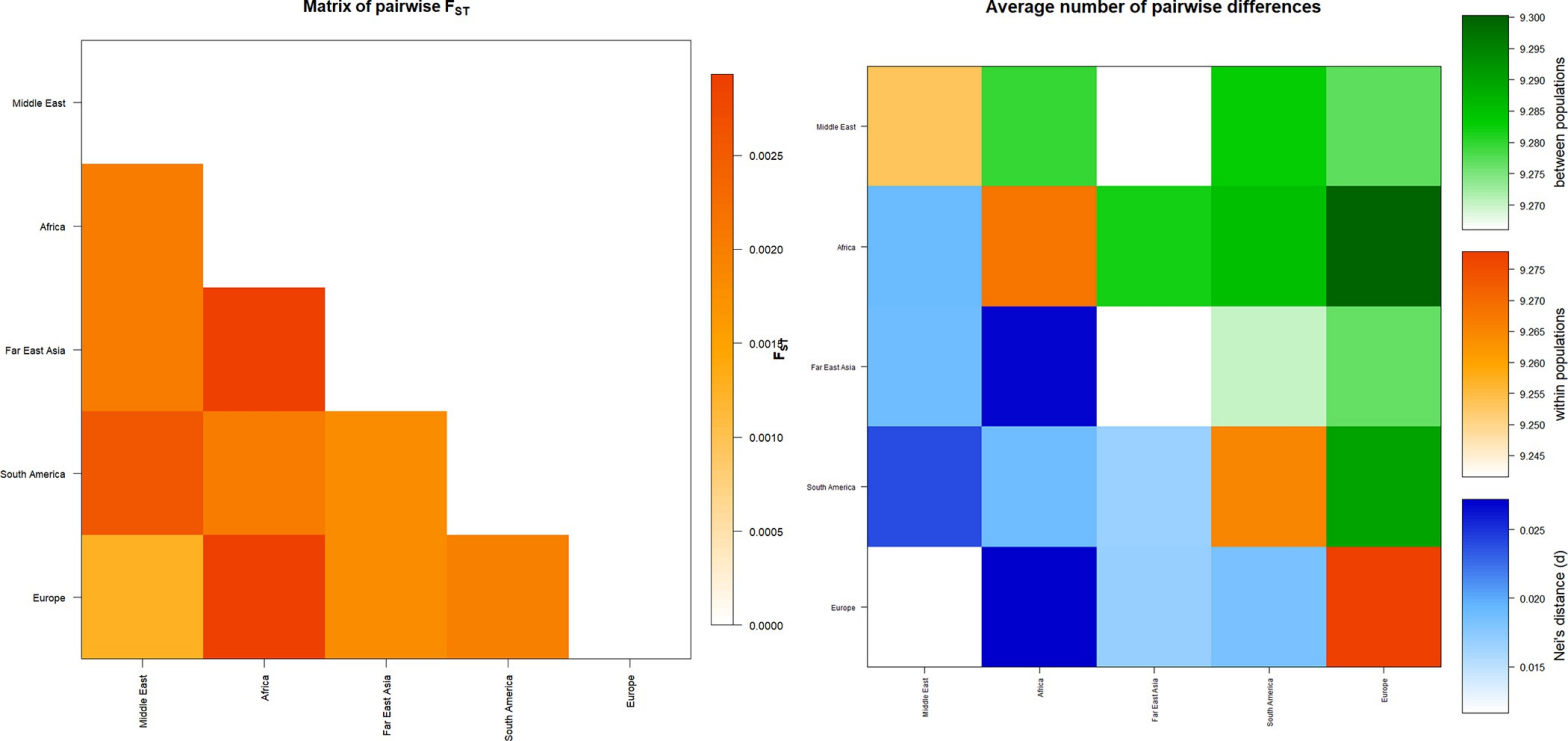
Plots displaying the average number of pairwise differences within and between the five continents. These matrices were generated using Arlequin 3.5.2.2 software.

The continental pairwise matrix showed more differentiation between the Middle East and South America (F_ST_ = 0.00257) compared to the differentiation between the Middle East and Africa (F_ST_ = 0.00206), Far East Asia (F_ST_ = 0.00203) and Europe (F_ST_ = 0.00125), as shown in Fig 5A and S9 Table (S1 File). Pairwise F_ST_ and the average pairwise differences between and within populations in different continents, in addition to the corrected average pairwise difference between populations of the five different continents were calculated. Nei’s distance results showed that the highest genetic distance was between the Middle East and South America (0.02383) while the lowest genetic distance was between the Middle East and Europe (0.01162). The highest average number of pairwise differences within the population was in Europe (9.27788) and the lowest was in Far East Asia (9.24154). The highest corrected average pairwise difference was between Africa and Europe (9.30024) and the lowest was between Far East Asia and the Middle East (9.26607), as shown in Fig 5B and S9 Table in S1 File.

### Population admixtures and ancestry variability

**Fig 6:**
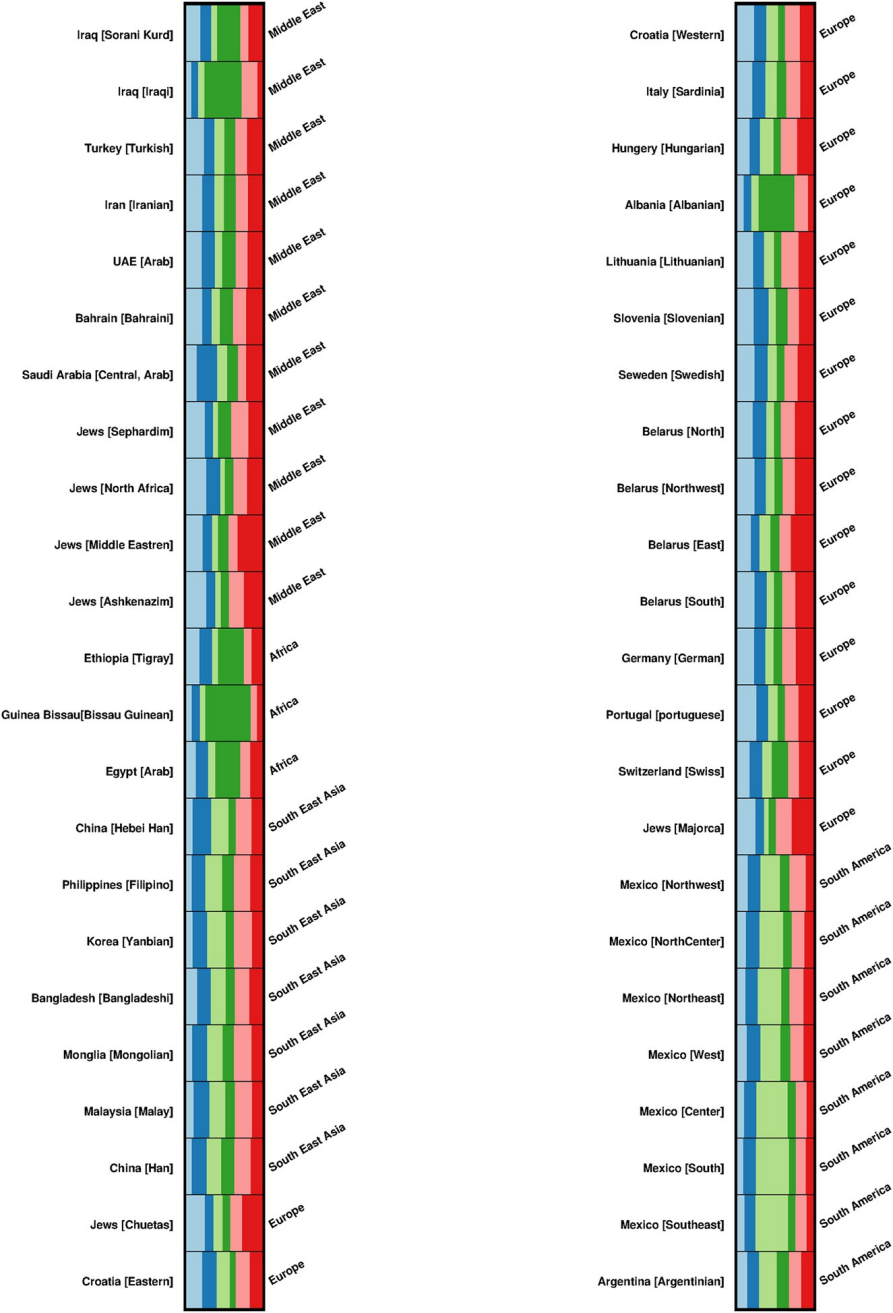
Population structure analysis using investigator Argus X-12 STR markers. The results show 6 clusters (K = 6) using 46 ethnic groups and populations from five different geographical regions (Europe, South America, the Middle East, Africa and South East Asia).

The structure of 46 ethnic groups and populations was investigated using the program STRUCTURE version 2.3.4 and an admixture model. The populations Q-matrices were created using X-STRs data from 12 markers across 46 populations (6,634 individuals). Six clusters have developed. The five geographical regions studied (Africa, the Middle East, South East Asia, Europe, and South America) did not reveal any distinct subpopulation genetic structures (Fig 6).

The FSTruct programme was used to analyse the $F_{ST}/F_{ST}^{max}$ ratio of the membership coefficient variability differences between admixed and non-admixed populations provided by the STRUCTURE analysis to investigate ancestry variability in the five geographical regions.

**Fig 7:**
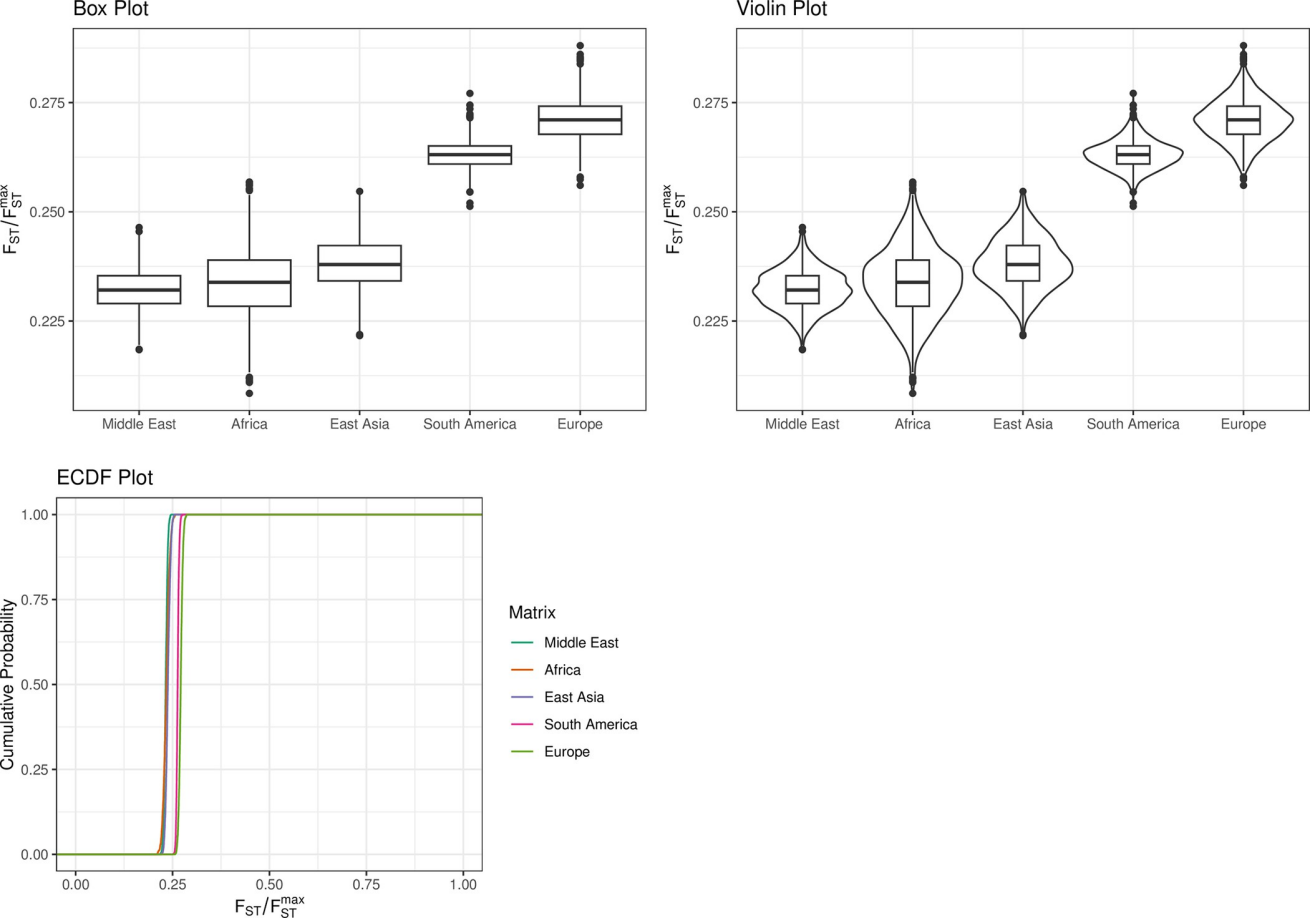
Box plot, violin plot and empirical cumulative distribution function (ECDF) plot of the bootstrap distribution of $F_{ST}/F_{ST}^{max}$ for each Q matrix in the STRUCTURE analysis.

The results showed that the $F_{ST}/F_{ST}^{max}$ ratio was lowest in the Middle Eastern and African populations, 0.23243 and 0.23395 respectively, while it was the highest in both the European and South American populations, 0.27122 and 0.26324 respectively, as shown in Fig 7 and S10 Table (S1 File).

Pairs of bootstrap distributions of $F_{ST}/F_{ST}^{max}$ were significantly different (p < 2e-16) for four pairwise combinations and P = 1.40E-08 between the Middle Eastern and the African populations, Wilcoxon rank-sum test. For all stats and ratios, and all stats and matrices, the Kruskal-Wallis chi-squared showed that there are significant differences between the groups being compared in the study (3921, df = 4, p-value = 2.2e-16).

### Allelic richness in different population groups

**Fig 8:**
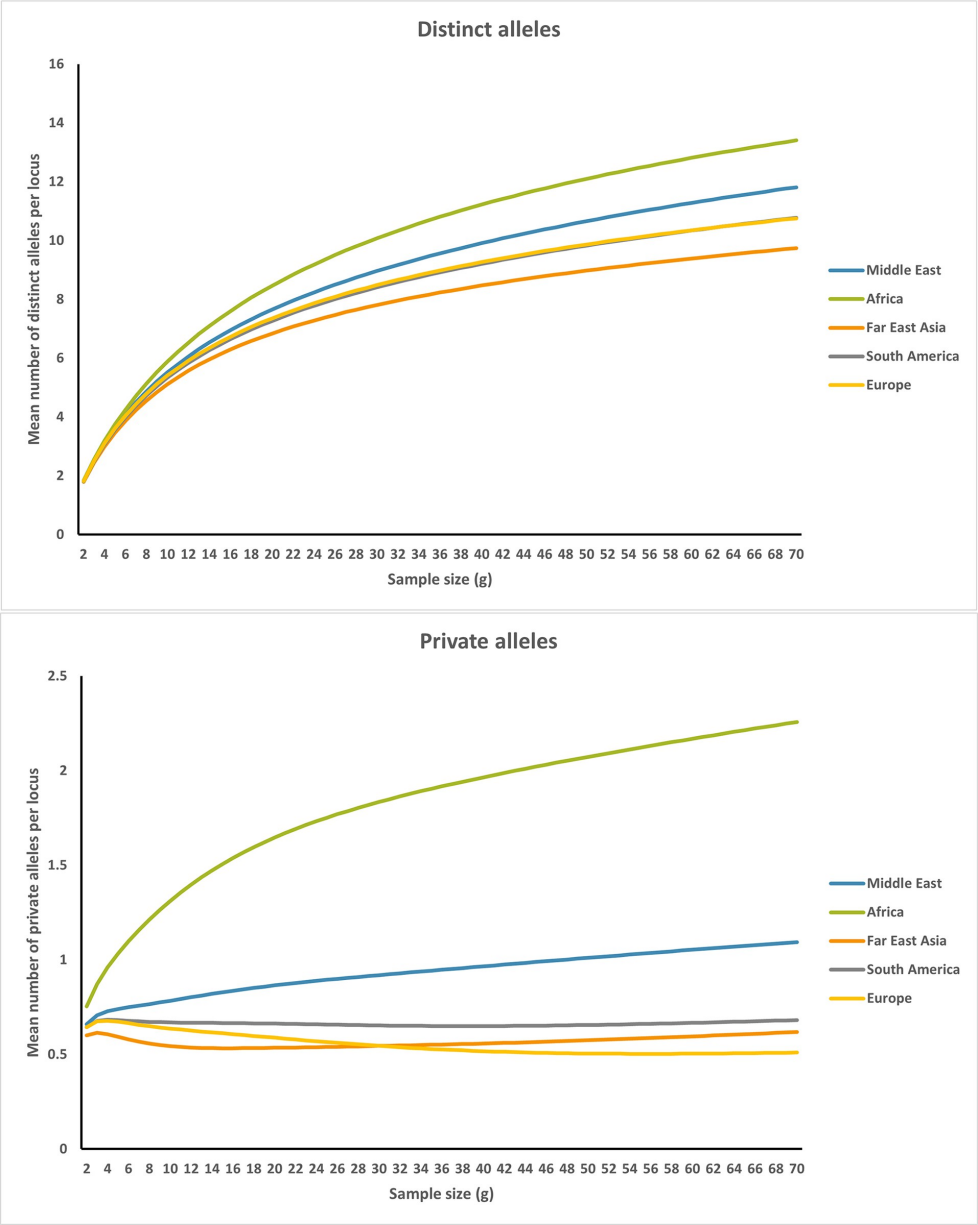
The mean number of (A) distinct alleles per locus and (B) private alleles per locus of the five regions: Middle East, Africa, Far East Asia, South America and Europe.

This study examined two allelic richness parameters, specifically distinct and private alleles, across five distinct geographical regions. The findings revealed that the distinct alleles demonstrated similarities between Europe and South America, while displaying the highest levels in Africa and the Middle East, and the lowest levels in Far East Asia (Fig 8A, S11A Table (S1 File)). Moreover, the analysis revealed that Africa and the Middle East exhibited the highest levels of private alleles, while Europe and Far East Asia displayed the lowest levels (Fig 8B, S11B Table (S1 File)).

## Discussion

The Argus X-12 (Qiagen, Hilden, Germany) is the most often used X-STRs kit in the field of forensic genetics. However, because the 12 X-STRs markers in this kit were largely characterised for European ancestry, several of the genetic variants observed in other population groups were missed out. To address this issue, the Argus X-12 QS emerged in the forensic field as an optimised version containing the same markers [1].

There has been few genetic research on the Iraqi Kurdish population, with the majority of previous work focusing on Y-STRs, mtDNA, and autosomal STRs [9, 10, 65, 66]. By analysing X chromosome markers, this research study hopes to contribute to a better knowledge of genetic diversity among the Kurdish population. Previous research in this field was limited, with only one study analysing the genetic makeup of the Kurdish population utilising only five X-STRs markers and treating the entire Kurdish population as a single group for comparison analysis [67]. In contrast, this study takes a broader approach, evaluating twelve X-STRs markers and focused specifically on a subgroup of the Kurdish community, the Iraqi Sorani Kurds.

The DXS10135 locus in the Kurdish population exhibited the highest polymorphism information content (PIC), which is comparable to Bahrain, UAE, Jews, Iraq, Lithuania, Turk, Slovenia, Albania, Guinea-Bessau, Filipino, Mongolia, Eastern Han, Hebei Han, Croatia, and Argentina [30–32, 34, 37, 40, 43, 44, 49, 54]. The lowest PIC was found at the locus DXS8378, which is shared by Argentina, Croatia, Turkey, Slovenia, and Albania, whereas the lowest PIC was found among other groups at the locus DXS7423. The bi-allele marker was observed in one male at the locus DXS7423; a doubled allele at the same locus was previously observed in the Iraqi population [34].

A rare allele was observed in two individuals, which is not presented in the allelic ladder of the kit at locus DXS8378 (allele = 6). This off-ladder allele at the same locus, DXS8378, was reported in the earlier study of the German population [47]. In addition, a null allele was also observed in one sample at the locus DXS10079 which, to our knowledge, has not been previously reported in other populations.

When MEC values were considered, the forensic efficacy of X-STRs in complex scenarios including kinship and paternity was obvious. These MEC calculations proved crucial in the examination of complex kinship and paternity scenarios, especially when the reference DNA sample came from the child’s putative grandmother, the father’s mother, rather than the alleged father.

The phylogenic tree showed that the Sorani Kurds, Saudi Arabia, Turkey, Iran, Ethiopia and Argentina fell into one cluster with the European populations. This study confirms the findings of a previous mtDNA study of the Sorani Kurds, which found that they are genetically related to the European lineage [65]. Furthermore, based on X-STRs, the Bahraini and Emirati populations were more closely linked, supporting the findings of Al-Snan’s previous study [30].

Even after applying Bonferroni’s correction, the linkage disequilibrium tests revealed one significant result between the DXS10103 and DXS10101 locus pairs from LG 3. A similar result with statistically substantial LD between DXS10103 and DXS10101 has previously been observed in the Iranian, Filipino, Mongolian, and Chinese Han populations [35, 40, 43, 44]. Another study found significant associations between the markers DXS10103 and DXS10101, as well as DXS10103 and HPRTB in the Chuetas Jewish population [32]. However, various population studies have found substantial levels of LD for various pairs of loci within different linkage groups [30, 31, 34, 37, 49, 54]. The observed variations are likely attributable to the sample size, since detecting linkage disequilibrium typically requires a substantial number of samples [68].

In comparison to the Y chromosome, which demonstrated a high level of gene diversity across all PPY23 kits in a global analysis [69], this study demonstrated a low degree of gene diversity across all 12 X-STRs in the Investigator Argus X-12 QS kit (Qiagen) panel.

Unlike the Y chromosome, the X chromosome population Q-matrices did not reveal a distinct subpopulation genetic structure [11]. In light of the findings, it can be concluded that population admixture did not exhibit significant relevance in the context of subpopulation clusters. Consequently, the determination of the specific geographical region to which the Sorani Kurds can be attributed within the five primary regions investigated in this study proved to be unfeasible. This could be explained by the fact that the X chromosome differs from the Y chromosome in that it undergoes recombination. The entirety of the Y chromosome functions as a single locus and has a single genealogical history. On the other hand, the X chromosome gets broken by recombination every generation, giving different regions on the chromosome different histories [3].

Individuals within populations that have recently undergone admixture differ substantially in terms of their ancestry, with some individuals deriving the majority of their ancestry from one source population and others from a different one. As a result, it might be hypothesised that admixed populations produce more ancestry variability than nonadmixed populations when assessing inferred cluster memberships. This was clearly shown in this study, as the Middle Eastern and African populations had the lowest ancestral variability as compared to European and South American populations.

Based on numerous viewpoints on the history of human migrations, we can evaluate patterns of distinct and private allelic richness in relation to our expectations. The higher number of distinct and private alleles in Africa corresponds to the pattern expected for models of human evolution that began in Africa and spread to other regions through a succession of founder events. The combination of the physically contiguous regions of Africa and the Middle East contains the greatest number of alleles. Since many alleles in the founding population would have only moved along part of their migration outside of Africa. The findings of this study on allelic richness and ancestry variability are consistent with the predictions of African-origin models that include serial founder effects throughout outward migrations [70].

The main limitation encountered in this research endeavour was the limited availability of X-STRs data. As seen, this study only included three African countries and two South American countries, necessitating additional data to enhance the understanding of the X-STRs population genetics.

In conclusion, this study represents the pioneering investigation into the X-STRs data pertaining to the population of Iraqi Sorani Kurds. The establishment of a comprehensive population database has enabled the utilization of STR markers included in the Argus X-12 Kit in routine forensic casework. The remarkable statistical parameters exhibited by these loci, in conjunction with their independent inheritance patterns, render them an invaluable marker set for both forensic casework and population genetics research. The use of X-STRs loci may prove to be a significant complement to autosomal and Y-STRs markers, particularly in complicated kinship and paternity instances.

## Supporting information

S1 File S1 Table. Haplotype frequencies for 12 X‐STRs markers in four linkage groups (LGs), N = 117. LG1 = (DXS8378, DXS10135, DXS10148), LG2 = (DXS7132, DXS10074, DXS10079), LG3 = (DXS10101, DXS10103, HPRTB), LG4 = (DXS7423, DXS10134, DXS10146). S2 Table: Allele frequencies and sample size by locus of the Sorani Kurd. S3 Table: Forensic parameters, Number of alleles (Aall), Gene diversity (GD), polymorphism information content (PIC), match probability (PM), power of discrimination (PD) and Mean Exclusion Chance (MEC) of the Sorani Kurd males. S4 Table: Pairwise p values of linkage disequilibrium test for all pairs of loci using 117 male samples from Sorani Kurdish population. S5 Table: Forensic parameters, Gene diversity (GD), polymorphism information content (PIC), match probability (PM) and power of discrimination (PD), of the five geographical regions Middle East, Africa, Far East Asia, Europe and South America. S6 Table: Pairwise genetic distances (Fst) based on haplotypes of 36 global populations. S7 Table: Average number of pairwise differences using data from 36 populations. Above diagonal: Average number of pairwise differences between populations (PiXY), Diagonal elements: (yellow highlighted): Average number of pairwise differences within population (PiX), Below diagonal: Corrected average pairwise difference (PiXY-(PiX+PiY)/2). S8 Table: Pairwise genetic distances based on F_ST_ between the five continents. S9 Table: average number of pairwise differences (F_ST_) within and between continents. Above diagonal: Average number of pairwise differences between populations (PiXY), Diagonal elements (yellow highlighted): Average number of pairwise differences within population (PiX), Below diagonal: Corrected average pairwise difference (PiXY-(PiX+PiY)/2). S10 Table: (A) Q statistics for the five poulations Middle East, Africa, Far East Asia and South America. (B) Pairwise comparisons using Wilcoxon rank sum test with continuity correction. S11 Table: distinctive alleles and private alleles on a continental scale: Middle East, Africa, Far East Asia, South America and Europe. (A) Distinctive alleles, (B) Private alleles.
(XLSX)

S2 File S1 Fig. Plot distribution of allele frequencies per locus using Investigator Argus X-12 QS kit. S2 Fig. Electropherogram shows variant alleles in three samples of the Sorani Kurd males. (A) An off-ladder allele was observed at locus DXS8378 (allele = 6). (B) Duplicated alleles (14, 15) were found at locus DXS7423. (C) A null allele was observed at locus DXS10079. S3 Fig. P-values for the pairwise exact test matrix for linkage disequilibrium among the 12 X-STR loci. S4 Fig. Ranking of Investigator Argus X-12 QS kit (Qiagen) markers by gene diversity (GD). Rank within continental residency groups, i.e. Middle East (n = 1224), Africa (n = 448), Far East Asia (n = 815), South America (n = 1167) and Europe (n = 2980).
(DOCX)
