# Supplementary material for: An X-STRs analysis of the Iraqi Sorani Kurds
Source: PLoS One. 2023 Nov 27;18(11):e0294973. doi: 10.1371/journal.pone.0294973 (PMC10681225; doi:10.1371/journal.pone.0294973)
Supplement: S2 File — S1 Fig. Plot distribution of allele frequencies per locus using Investigator Argus X-12 QS kit. S2 Fig. Electropherogram shows variant alleles in three samples of the Sorani Kurd males. (A) An off-ladder allele was observed at locus DXS8378 (allele = 6). (B) Duplicated alleles (14, 15) were found at locus DXS7423. (C) A null allele was observed at locus DXS10079. S3 Fig. P-values for the pairwise exact test matrix for linkage disequilibrium among the 12 X-STR loci. S4 Fig. Ranking of Investigator Argus X-12 QS kit (Qiagen) markers by gene diversity (GD). Rank within continental residency groups, i.e. Middle East (n = 1224), Africa (n = 448), Far East Asia (n = 815), South America (n = 1167) and Europe (n = 2980). (DOCX) [file pone.0294973.s002.docx]

**Supplementary figures**


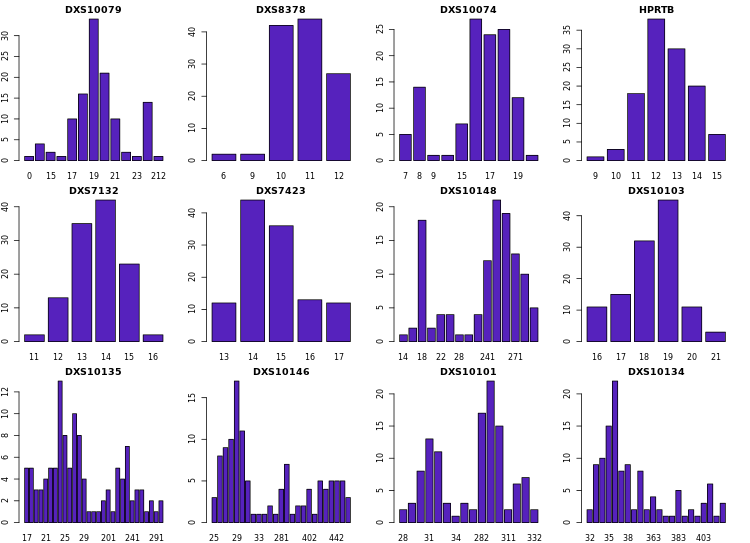


S1 Fig. Plot distribution of allele frequencies per locus using Investigator Argus X-12 QS kit.

| A | 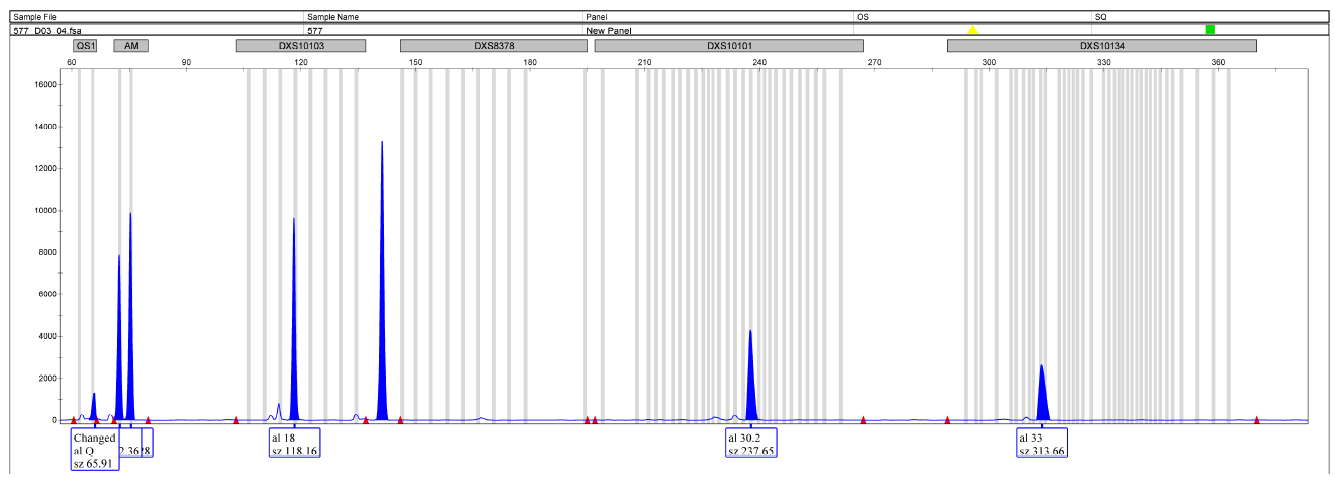 |
| --- | --- |
| B | 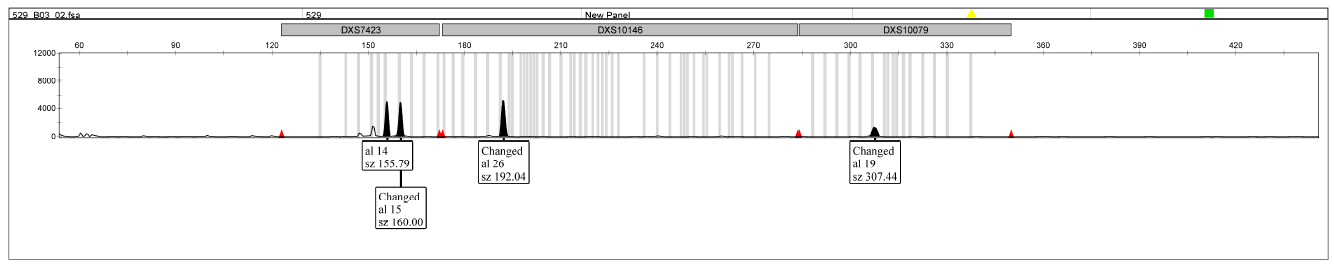 |
| C | 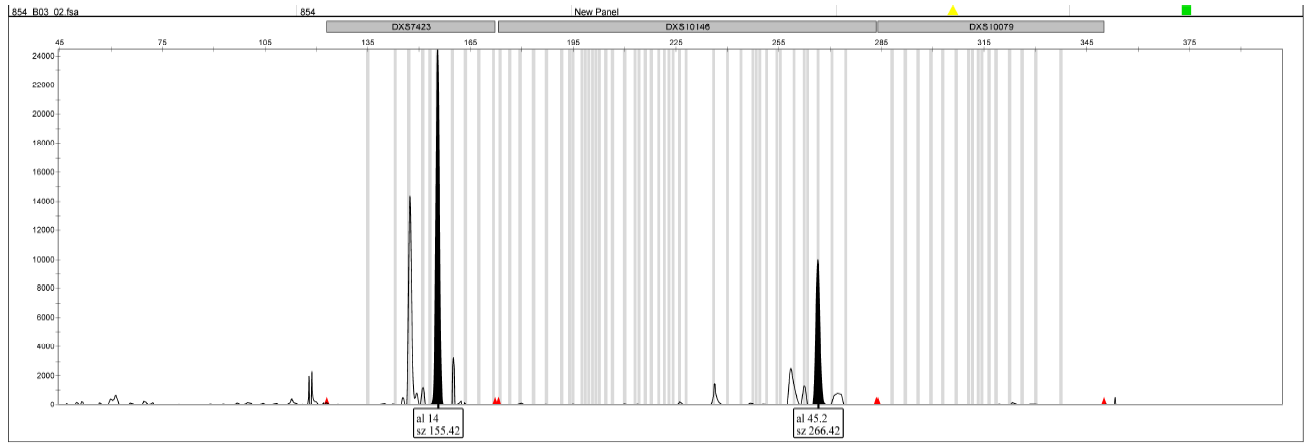 |

S2 Fig. Electropherogram shows variant alleles in three samples of the Sorani Kurd males. (A) An off-ladder allele was observed at locus DXS8378 (allele = 6). (B) Duplicated alleles (14, 15) were found at locus DXS7423. (C) A null allele was observed at locus DXS10079.


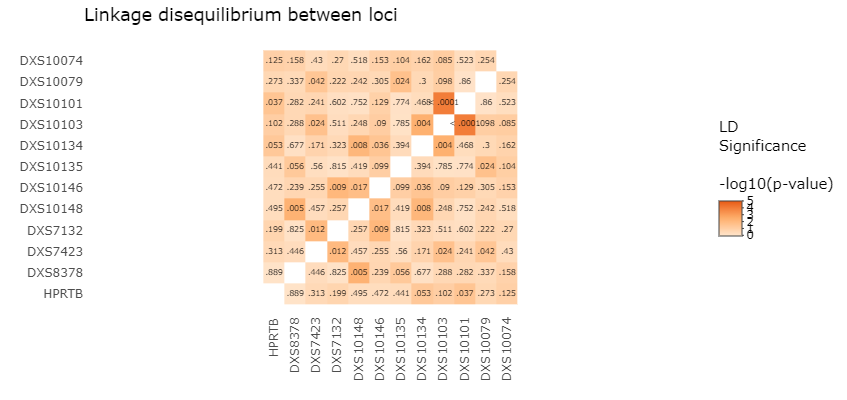


S3 Fig. P-values for the pairwise exact test matrix for linkage disequilibrium among the 12 X-STR loci.

S4 Fig. Ranking of Investigator Argus X-12 QS kit (Qiagen) markers by gene diversity (GD). Rank within continental residency groups, i.e. Middle East (n=1224), Africa (n = 448), Far East Asia (n = 815), South America (n=1167) and Europe (n = 2980).
